# Electronic and Structural Properties of *AB*O_3_: Role of the *B*-O Coulomb Repulsions for Ferroelectricity

**DOI:** 10.3390/ma4010260

**Published:** 2011-01-17

**Authors:** Kaoru Miura, Masaki Azuma, Hiroshi Funakubo

**Affiliations:** 1 Corporate R&D Headquarters, Canon Inc., 3-30-2 Shimomaruko, Ohta-ku, Tokyo 146-8501, Japan; 2 Materials and Structures Laboratory, Tokyo Institute of Technology, 4259 Nagatsuta, Midori-ku, Yokohama 226-8502, Japan; 3 Department of Innovative and Engineered Materials, Tokyo Institute of Technology, 4259 Nagatsuta, Midori-ku, Yokohama 226-8502, Japan

**Keywords:** ferroelectrics, electronic band structure, phase transitions, coulomb repulsion

## Abstract

We have investigated the role of the Ti–O Coulomb repulsions in the appearance of the ferroelectric state in BaTiO${}_{3}$ as well as the role of the Zn–O Coulomb repulsions in BiZn${}_{0.5}$Ti${}_{0.5}$O${}_{3}$, using a first-principles calculation with optimized structures. In tetragonal BaTiO${}_{3}$, it is found that the Coulomb repulsions between Ti 3s and 3p states and O 2s and 2p states have an important role for the appearance of Ti ion displacement. In BiZn${}_{0.5}$Ti${}_{0.5}$O${}_{3}$, on the other hand, the stronger Zn–O Coulomb repulsions, which are due to the 3s, 3p, and 3d (d${}^{10}$) states of the Zn ion, have more important role than the Ti–O Coulomb repulsions for the appearance of the tetragonal structure. Our suggestion is consistent with the other ferroelectric perovskite oxides ABO${}_{3}$ in the appearance of tetragonal structures as well as rhombohedral structures.

## 1. Introduction

Since Cohen [1,2] proposed an origin for ferroelectricity in perovskite oxides, investigations of ferroelectric materials using first-principles calculations have been extensively studied [3,4,5,6,7,8,9,10,11,12,13,14,15,16,17,18,19,20,21]. Currently, using the pseudopotential (PP) methods, most of the crystal structures in ferroelectric perovskite oxides (ABO${}_{3}$) as well as perovskite-related oxides can be precisely predicted. However, it is also known that the most stable structures of ABO${}_{3}$ optimized by the first-principles PP methods are sometimes inconsistent with the experimental results.

BaTiO${}_{3}$ is a well-known ferroelectric ABO${}_{3}$, and shows the tetragonal structure at room temperature. However, even in this well-known material, the optimized structure by the PP methods of first-principles calculations is strongly dependent on the choice of the Ti PPs, *i.e*., preparation for Ti 3s and 3p semicore states in addition to Ti 3d and 4s valence states is essential to the appearance of the tetragonal structure. This is an important problem for ferroelectricity, but it has been generally recognized for a long time that this problem is within an empirical framework of the calculational techniques [22]. It is known that ferroelectric state appears when the long-range forces due to the dipole-dipole interaction overcome the short-range forces due to the Coulomb repulsions. Cohen [1,2] proposed that the hybridization between Ti 3d state and O 2p state (Ti 3d–O 2p) in BaTiO${}_{3}$ and PbTiO${}_{3}$, which weakens the short-range force of the Coulomb repulsions between Ti and O ions, is the origin of ferroelectricity. However, it seems to be difficult to consider explicitly whether the long-range force due to the dipole-dipole interaction can or cannot overcome the short-range force only with the Ti 3d–O 2p hybridization. Investigations about the relationship between the Ti-O Coulomb repulsions and the appearance of ferroelectricity were separately reported. Theoretically, we previously investigated [15] the influence of the Ti–O${}_{z}$ Coulomb repulsions on Ti ion displacement in tetragonal BaTiO${}_{3}$ and PbTiO${}_{3}$, where O${}_{z}$ denotes the O atom to the *z*-axis (Ti is displaced to the *z*-axis). Whereas the hybridization between Ti 3d state and O${}_{z}$ 2p${}_{z}$ state stabilize Ti ion displacement, the strong Coulomb repulsions between Ti 3s and 3p${}_{z}$ states and O 2p${}_{z}$ states do not favorably cause Ti ion displacement. Experimentally, on the other hand, Kuroiwa *et al.* [23] showed that the appearance of ferroelectric state is closely related to the total charge density of Ti–O bondings in BaTiO${}_{3}$. As discussed above, investigation about a role of Ti 3s and 3p states is important in the appearance of the ferroelectric state in tetragonal BaTiO${}_{3}$ .

Generally, it has been known that the most stable structure of ABO${}_{3}$ is closely related to the tolerance factor *t*,

(1) $$t\equiv \frac{r_{A}+r_{O}}{\sqrt{2}\;\left(\;r_{B}+r_{O}\right)}\;,$$

where $r_{A}$, $r_{B}$, and $r_{O}$ denote the ionic radii of *A*, *B*, and O ions, respectively. In general ferroelectric ABO${}_{3}$, the most stable structure is tetragonal for t≳ 1, cubic for t≈ 1, and rhombohedral or orthorhombic for t≲ 1 [19,24]. In fact, both BaTiO${}_{3}$ with *t* = 1.062 and PbTiO${}_{3}$ with *t* = 1.019 show tetragonal structures in room temperature. However, recently, BiZn${}_{0.5}$Ti${}_{0.5}$O${}_{3}$ (BZT) with *t* = 0.935 has experimentally been observed [25] to show a tetragonal PbTiO${}_{3}$-type structure with random Zn/Ti ordering and high c/a ratio (1.211). This result is in contrast to that of BiMg${}_{0.5}$Ti${}_{0.5}$O${}_{3}$ (BMT) with *t* = 0.939, *i.e*., the most stable structure was reported to be the orthorhombic [26] or rhombohedral structure [27]. Theoretically, Qi *et al.* [9] reported the calculated results of BZT by a first-principles PP method. They optimized the tetragonal and rhombohedral structures of BZT with ten configurations of Zn/Ti ordering and concluded that the most stable structure is the tetragonal structure with Zn and Ti planes stacking alternately perpendicular to the tetragonal direction. However, their conclusion is inconsistent with the experimental result [25] of the most stable tetragonal structure with random Zn/Ti ordering. Wang *et al.* [10] also reported the first-principles calculational results of BZT. They emphasized the importance of the strong Bi–O hybridization as well as the Ti–O and Zn–O ones for the appearance of the tetragonal structure with high c/a ratio. However, the detail relationship between their strong hybridizations and the appearance of the tetragonal structure seem not to be discussed. Therefore, investigation about a role of the Zn–O Coulomb repulsion, in addition to a role of the Ti–O Coulomb repulsion, is important in the appearance of the ferroelectric state in tetragonal BZT .

Recently, we investigated the roles of the Ti–O and Zn–O Coulomb repulsions in the appearance of a ferroelectric states in tetragonal BaTiO${}_{3}$ [17] and BZT [18] by the analysis of a first-principles PP method. Our investigation suggested that the Coulomb repulsions between Ti 3s and 3p${}_{x(y)}$ states and O${}_{x(y)}$ 2*s* and 2p${}_{x(y)}$ states have an important role in the appearance of the ferroelectric state in tetragonal BaTiO${}_{3}$. Moreover, in tetragonal BZT, the Coulomb repulsions between Zn 3s, 3p, and 3d (d${}^{10}$) states and O 2*s* and 2p states, in addition to the above Ti–O Coulomb repulsions, have another important role in the appearance of the ferroelectric state. In this manuscript, we discuss a general role of *B*–O Coulomb repulsions for ferroelectricity in tetragonal ABO${}_{3}$ based on our previous reports [17,18].

## 2. Calculations

**Figure 1:**
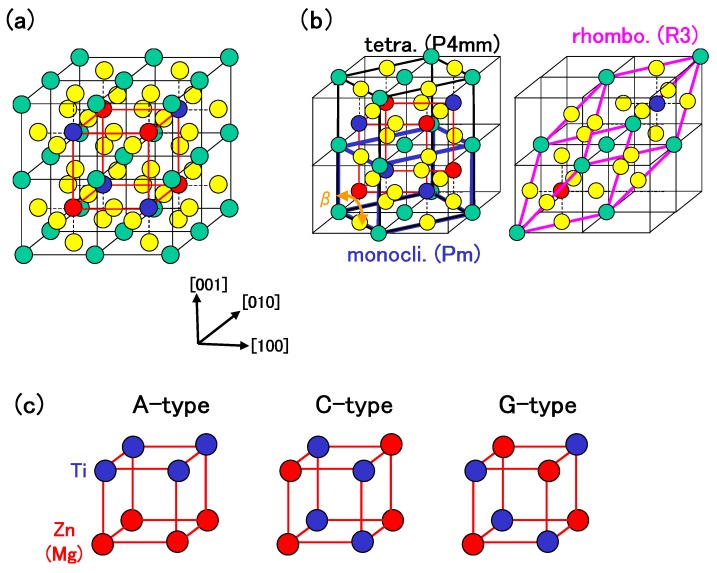
(**a**) 2 × 2 × 2 super cell of BZT (or BMT). Green, yellow, red, and blue balls denote Bi, O, Zn (or Mg), and Ti atoms, respectively. The lattice with red lines denotes that of Zn (or Mg) and Ti atoms in a tetragonal structure, whose configuration is illustrated in (c); (**b**) Unit cells of (C-type) tetragonal, monoclinic, and rhombohedral structures; (**c**) Configurations of Zn (or Mg) and Ti atoms in A-, C-, and G-type tetragonal structures [18].

Calculations of BaTiO${}_{3}$, BZT and BMT were performed using the abinit package code [28], which is one of the norm-conserving PP (NCPP) methods. Electron-electron interaction was treated in the local-density approximation (LDA) [29]. Pseudopotentials were generated using the opium code [30]:

zw (i) In BaTiO${}_{3}$, 5s, 5p and 6s electrons for Ba PP, and 2s and 2p electrons for O PP were treated as semicore or valence electrons, respectively. Moreover, in order to investigate the role of Ti 3s and 3p states, two kinds of Ti PPs were prepared: one is the Ti PP with 3s, 3p, 3d and 4s electrons treated as semicore or valence electrons (Ti3spd4s PP), and the other is the Ti PP with only 3d and 4s electrons treated as valence electrons (Ti3d4s PP). In both PPs, the differences between the calculated result and the experimental one are within 1.5 % of the lattice constant and within 10 % of the bulk modulus in the optimized calculation of bulk Ti. The cutoff energy for plane-wave basis functions was set to be 50 Hartree (Hr). A 6 × 6 × 6 Monkhorst-Pack *k*-point mesh was set in the Brillouin zone of the unit cell. The number of atoms in the unit cell was set to be five, and positions of all the atoms were optimized within the framework of the tetragonal (P4mm) or rhombohedral (R3m) structure.

zw (ii) In BZT and BMT, 5d, 6s, and 6p electrons for Bi PP, and 2s and 2p electrons for O PP were considered as semicore and valence electrons, respectively. Moreover, in order to investigate the roles of Zn and Ti 3s and 3p states, and Mg 2s and 2p states, two types of PPs were prepared: the PPs with only Zn and Ti 3d and 4s states, and Mg 3s states, considered as valence electrons (Case I), Zn and Ti 3s, 3p, 3d, and 4s states, and Mg 2s, 2p, and 3s states considered as semicore or valence electrons (Case II). The cutoff energy for plane-wave basis functions was set to be 70 Hr for Case I and 110 Hr for Case II. A 4 × 4 × 4 Monkhorst-Pack *k*-point mesh was set in the Brillouin zone of the unit cell. The calculated results can be discussed within 0.02 eV per formula unit (f.u.) using the above conditions. The present calculations were performed for the monoclinic, rhombohedral, and A-, C-, and G-type tetragonal structures. The number of atoms in the unit cell was set to be 10 for the rhombohedral and monoclinic structures, and 20 for the A-, C-, and G-type tetragonal structures. The structures of the above unit cells are illustrated in Figure 1(a)–Figure 1(c). Positions of all the atoms were optimized within the framework of the rhombohedral (R3), monoclinic (Pm), and tetragonal (P4mm) structures.

## 3. Results and Discussion

### 3.1. BaTiO${}_{3}$: Role of Ti 3s and 3p states for ferroelectricity

In this subsection, we discuss the role of Ti 3s and 3p states for ferroelectricity for ferroelectricity in tetragonal BaTiO${}_{3}$.

**Figure 2:**
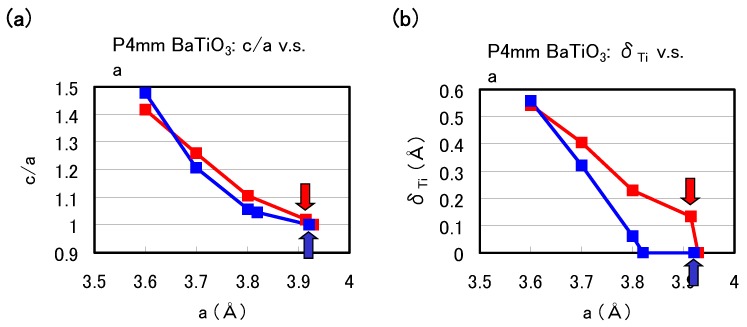
Optimized calculated results as a function of *a* lattice constants in tetragonal BaTiO${}_{3}$: (**a**) c/a ratio and (**b**) $\delta_{\mathrm{Ti}}$ to the [001] axis. Red lines correspond to the results with the Ti3spd4s PP, and blue lines correspond to those with the Ti3d4s PP. Results with arrows are the fully optimized results, and the other results are those with *c* and all the inner coordinations optimized for fixed *a* [17].

Figure 2(a) and Figure 2(b) show the optimized results for the ratio *c*/*a* of the lattice parameters and the value of the Ti ion displacement ($\delta_{\mathrm{Ti}}$) as a function of the *a* lattice constants in tetragonal BaTiO${}_{3}$, respectively. Results with arrows are the fully optimized results, and the others results are those with the *c* lattice constants and all the inner coordinations optimized for fixed *a*. Note that the fully optimized structure of BaTiO${}_{3}$ is tetragonal with the Ti3spd4s PP, whereas it is cubic ($Pm\bar{3}m$) with the Ti3d4s PP. As shown in Figure 2(a) and Figure 2(b), *c*/*a* and $\delta_{\mathrm{Ti}}$ show significantly different results for a≳ 3.7 Å whereas they show almost the same results for a≲ 3.7 Å, for both Ti PPs. This result suggests that the optimized results of ABO${}_{3}$ with smaller lattice constants, e.g., under high pressure [8], are almost independent of the choice of PP.

**Figure 3:**
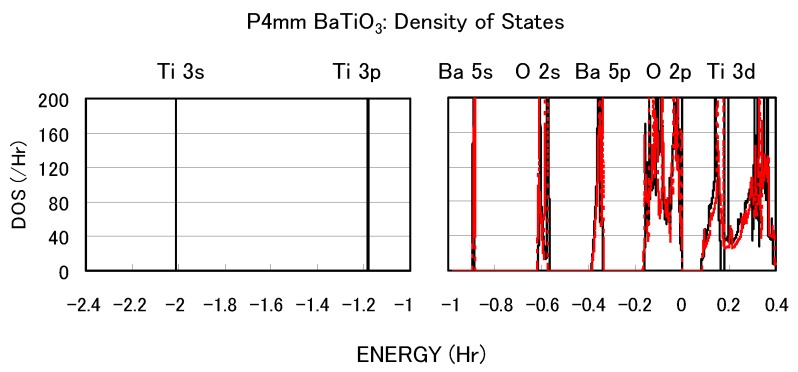
Total density of states (DOS) of fully optimized tetragonal BaTiO${}_{3}$ with the Ti3spd4s PP (solid line) and cubic BaTiO${}_{3}$ with the Ti3d4s PP (red dashed line) [17].

The calculated results shown in Figure 2 suggest that the explicit treatment of Ti 3s and 3p semicore states is essential to the appearance of ferroelectric states in BaTiO${}_{3}$. In the following, we investigate the role of Ti 3s and 3p states for ferroelectricity from two viewpoints. One viewpoint concerns hybridizations between Ti 3s and 3p states and other states. Figure 3 shows the total density of states (DOS) of tetragonal BaTiO${}_{3}$ with two Ti PPs. Both results are in good agreement with previous calculated results [11,12] by the full-potential linear augmented plane wave (FLAPW) method. In the DOS with the Ti3spd4s PP, the energy “levels", not bands, of Ti 3s and 3p states, are located at −2.0 Hr and −1.2 Hr, respectively. This result suggests that the Ti 3s and 3p orbitals do not make any hybridizations but only give Coulomb repulsions with the O orbitals as well as the Ba orbitals. In the DOS with the Ti3d4s PP, on the other hand, the energy levels of Ti 3s and 3p states are not shown because Ti 3s and 3p states were treated as the core charges. This result means that the Ti 3s and 3p orbitals cannot even give Coulomb repulsions with the O orbitals as well as the Ba orbitals.

**Figure 4:**
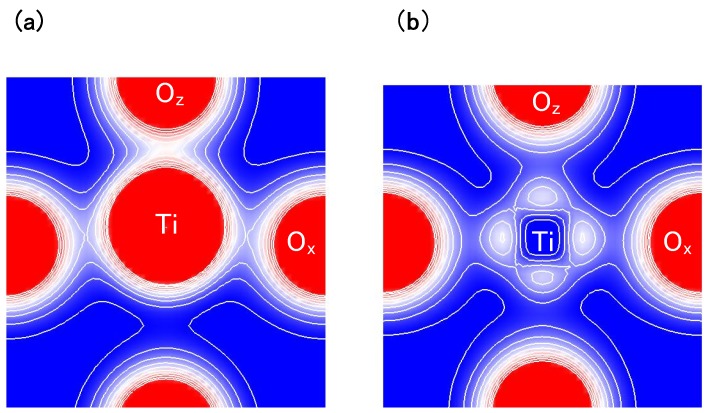
Two-dimensional electron-density contour map on the xz-plane for tetragonal BaTiO${}_{3}$: (**a**) with the Ti3spd4s PP; and (**b**) with the Ti3d4s PP. The optimized calculated results with *a* fixed to be 3.8Å are shown in both figures. The electron density increases as color changes from blue to red via white. Contour curves are drawn from 0.4 to 2.0 *e*/${Å}^{3}$ with increments of 0.2 *e*/${Å}^{3}$ [17].

Another viewpoint is about the Coulomb repulsions between Ti 3s and 3p${}_{x\;(y)}$ states and O${}_{x\;(y)}$ 2s and 2p${}_{x\;(y)}$ states in tetragonal BaTiO${}_{3}$. Figure 4(a) and Figure 4(b) show two-dimensional electron-density contour map on the xz-plane for tetragonal BaTiO${}_{3}$ with the Ti3spd4s PP, and that with the Ti3d4s PP, respectively. These are the optimized calculated results with *a* fixed to be 3.8 Å, and the electron density in Figure 4(a) is quantitatively in good agreement with the experimental result [23]. The electron density between Ti and O${}_{x}$ ions in Figure 4(a) is larger than that in Figure 4(b), which suggests that Ti ion displacement is closely related to the Coulomb repulsions between Ti 3s and 3p states and O 2s and 2p states along the [001] axis (the *z*-axis in this case).

The present discussion of the Coulomb repulsions is consistent with the previous reports. A recent soft mode investigation [13] of BaTiO${}_{3}$ shows that Ba ions contribute little to the appearance of Ti ion displacement along the [001] axis. This result suggests that Ti ion displacement is closely related to the structural distortion of TiO${}_{6}$ octahedra. In the present calculations, on the other hand, the only difference between BaTiO${}_{3}$ with the Ti3spd4s PP and with the Ti3d4s PP is the difference in the expression for the Ti 3s and 3p states, *i.e*., the explicit treatment and including core charges. However, our previous calculation [15] shows that the strong Coulomb repulsions between Ti 3s and 3p${}_{z}$ states and O${}_{z}$ 2s and 2p${}_{z}$ states do not favor Ti ion displacement along the [001] axis. This result suggests that the Coulomb repulsions between Ti 3s and 3p${}_{x\;(y)}$ states and O${}_{x\;(y)}$ 2s and 2p${}_{x\;(y)}$ states would contribute to Ti ion displacement along the [001] axis, and the suggestion is consistent with a recent calculation [14] for PbTiO${}_{3}$ indicating that the tetragonal and ferroelectric structure appears more favorable as the *a* lattice constant decreases.

**Figure 5:**
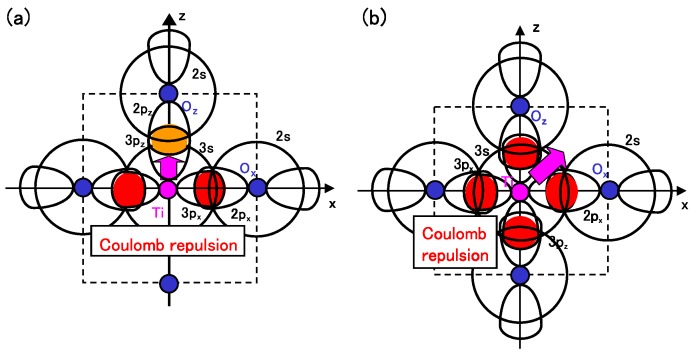
Illustrations of the proposed mechanisms for the Coulomb repulsions between Ti 3s and 3p states and O 2s and 2p states in BaTiO${}_{3}$: (**a**) anisotropic Coulomb repulsions between Ti 3s and 3p${}_{x\;(y)}$ states and O${}_{x\;(y)}$ 2s and 2p${}_{x\;(y)}$ states, and between Ti 3s and 3p${}_{z}$ states and O${}_{z}$ 2s and 2p${}_{z}$ states, in the tetragonal structure; (**b**) isotropic Coulomb repulsions between Ti 3s and 3p${}_{x\;(y)\;(z)}$ states and O${}_{x\;(y)\;(z)}$ 2s and 2p${}_{x\;(y)\;(z)}$ states, in the rhombohedral structure [17].

Considering the above investigations, we propose the mechanism of Ti ion displacement as follows: Ti ion displacement along the *z*-axis appears when the Coulomb repulsions between Ti 3s and 3p${}_{x\;(y)}$ states and O${}_{x\;(y)}$ 2s and 2p${}_{x\;(y)}$ states, in addition to the dipole-dipole interaction, overcome the Coulomb repulsions between Ti 3s and 3p${}_{z}$ states and O${}_{z}$ 2s and 2p${}_{z}$ states [15]. An illustration of the Coulomb repulsions is shown in Figure 5(a). In fully optimized BaTiO${}_{3}$ with the Ti3spd4s PP, the Ti ion can be displaced due to the above mechanism. In fully optimized BaTiO${}_{3}$ with the Ti3d4s PP, on the other hand, the Ti ion cannot be displaced due to the weaker Coulomb repulsions between Ti and O${}_{x\;(y)}$ ions. However, since the Coulomb repulsion between Ti and O${}_{z}$ ions in BaTiO${}_{3}$ with the Ti3d4s PP is also weaker than that in BaTiO${}_{3}$ with the Ti3spd4s PP, the Coulomb repulsions between between Ti and O${}_{x\;(y)}$ ions in addition to the log-range force become comparable to the Coulomb repulsions between Ti and O${}_{z}$ ions both in Ti PPs, as the lattice parameter *a* becomes smaller. The above discussion suggests that the hybridization between Ti 3d and O${}_{z}$ 2s and 2p${}_{z}$ stabilizes Ti ion displacement, but contribute little to a driving force for the appearance of Ti ion displacement.

It seems that the above proposed mechanism for tetragonal BaTiO${}_{3}$ can be applied to the mechanism of Ti ion displacement in rhombohedral BaTiO${}_{3}$, as illustrated in Figure 5(b). The strong isotropic Coulomb repulsions between Ti 3s and 3p${}_{x\;(y)\;(z)}$ states and O${}_{x\;(y)\;(z)}$ 2s and 2p${}_{x\;(y)\;(z)}$ states yield Ti ion displacement along the [111] axis. On the other hand, when the isotropic Coulomb repulsions are weaker or stronger, the Ti ion cannot be displaced and therefore it is favored for the crystal structure to be cubic.

**Figure 6:**
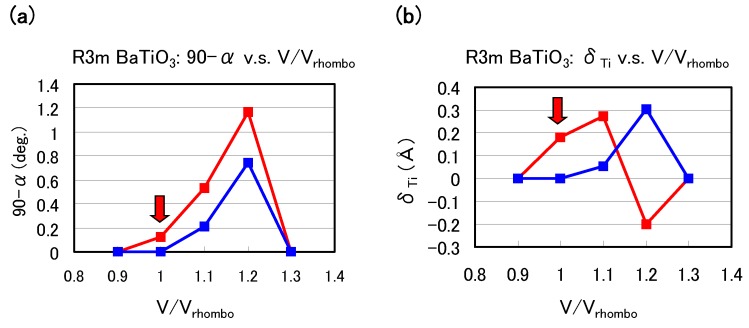
Optimized calculated results as a function of the fixed volumes of the unit cells in rhombohedral BaTiO${}_{3}$: (**a**) 90-α degree and (**b**) $\delta_{\mathrm{Ti}}$ to the [111] axis. Red lines correspond to the results with the Ti3spd4s PP, and blue lines correspond to those with the Ti3d4s PP. $V_{\mathrm{rhombo}}$ denote the volume of the fully optimized unit cell with the Ti 3spd4s PP. Results with arrows are the fully optimized results, and the other results are those with all the inner coordinations optimized for fixed volumes of the unit cells. Note that the Ti ion with the Ti3spd4s PP is oppositely displaced at $V_{\mathrm{rhombo}}/V$ = 1.2 [17].

Let us investigate the structural properties of rhombohedral BaTiO${}_{3}$. Figure 6(a) and Figure 6(b) show the optimized results of the 90-α degree and $\delta_{\mathrm{Ti}}$ as a function of fixed volumes of the unit cells in rhombohedral BaTiO${}_{3}$, respectively, where α denotes the angle between two lattice vectors. In these figures, α denotes the angle between two crystal axes of rhombohedral BaTiO${}_{3}$, and $\delta_{\mathrm{Ti}}$ denotes the value of the Ti ion displacement along the [111] axis. Results with arrows are the fully optimized results; $V_{\mathrm{rhombo}}$ denote the volume of the fully optimized unit cell with the Ti 3spd4s PP. The other results are those with all the inner coordinations optimized with fixed volumes of the unit cells. Our proposal mechanisms about the Coulomb repulsions seem to be consistent with the calculated results shown in Figure 6: For $V/V_{\mathrm{rhombo}}≲$ 0.9 or ≳ 1.3, the isotropic Coulomb repulsions are weaker or stronger, and the Ti ion cannot be displaced along the [111] axis and therefore the crystal structure is cubic for both Ti PPs. For 0.9 $≲V/V_{\mathrm{rhombo}}≲$ 1.3, on the other hand, the isotropic Coulomb repulsions are strong enough to yield Ti ion displacement for both Ti PPs. However, since the magnitude of the isotropic Coulomb repulsion is different in the two Ti PPs, the properties of the 90-α degree and $\delta_{\mathrm{Ti}}$ are different quantitatively.

### 3.2. BiZn${}_{0.5}$Ti${}_{0.5}$O${}_{3}$: Role of Zn 3s, 3p, and 3d states for ferroelectricity

As discussed in the previous subsection, the Coulomb repulsions between Ti 3s and 3p${}_{x\;(y)}$ states and O${}_{x\;(y)}$ 2s and 2p${}_{x\;(y)}$ states have an important role in the appearance of the ferroelectric state in tetragonal BaTiO${}_{3}$. In this subsection, we discuss the role of Zn 3d (d${}^{10}$) states in addition to 3s and 3p states for ferroelectricity in tetragonal BZT.

Table 1 shows a summary of the optimized results of BZT in Cases I and II. $\Delta E_{\mathrm{tot}}$ denotes the difference in total energy per f.u. between the rhombohedral and other structures. Although the lattice constant in each structure except the rhombohedral one seems to be quantitatively similar in both cases, properties of $\Delta E_{\mathrm{tot}}$ are different. In Case I, the rhombohedral structure is the most stable, which is in disagreement with the experimental result [25]. In Case II, on the other hand, the monoclinic structure, which is the “pseudo-C-type-tetragonal” structure, is the most stable. This result seems to be in disagreement with the experimental result, [25] but the calculated lattice constants are in good agreement with the experimental ones. Note that the magnitude of $\Delta E_{\mathrm{tot}}$ in Case II is markedly smaller than that in Case I. Table 2 shows a summary of the optimized results of BMT in Cases I and II. In contrast to BZT, the rhombohedral structure is the most stable structure in both cases, which is consistent with the experimental result [27]. Note that the magnitude of $\Delta E_{\mathrm{tot}}$ in Case II is comparable to that in Case I.

Table 1Summary of the optimized results of BZT in (a) Case I and (b) Case II. $\Delta E_{\mathrm{tot}}$ denotes the difference in total energy per f.u. between the rhombohedral and other structures [18].

**Table:** (a)

Structure	*a* (Å)	*c* (Å)	c/a	α (degree)	$\Delta E_{\mathrm{tot}}$ (eV/f.u.)
Tetragonal					
zwA-type	3.748	4.579	1.222	90	0.316
zwC-type	3.681	4.784	1.299	90	0.240
zwG-type	3.725	4.574	1.228	90	0.158
Monoclinic	3.735	4.741	1.269	β = 91.5	0.193
Rhombohedral	5.560		1	59.93	0
Experiment (ref. [25])	3.822	4.628	1.211	90

**Table:** (b)

Structure	*a* (Å)	*c* (Å)	c/a	α (degree)	$\Delta E_{\mathrm{tot}}$ (eV/f.u.)
Tetragonal					
zwA-type	3.711	4.662	1.256	90	0.135
zwC-type	3.670	4.789	1.305	90	0.091
zwG-type	3.684	4.698	1.275	90	0.047
Monoclinic	3.726	4.740	1.272	β = 91.1	−0.021
Rhombohedral	5.590		1	59.90	0
Experiment (ref. [25])	3.822	4.628	1.211	90	

Table 2Summary of the optimized results of BMT in (a) Case I and (b) Case II. $\Delta E_{\mathrm{tot}}$ denotes the difference in total energy per f.u. between the rhombohedral and other structures [18].

**Table:** (a)

Structure	*a* (Å)	*c* (Å)	c/a	α (degree)	$\Delta E_{\mathrm{tot}}$ (eV/f.u.)
Tetragonal					
zwA-type	3.788	4.260	1.125	90	0.397
zwC-type	3.707	4.586	1.237	90	0.357
zwG-type	3.758	4.348	1.157	90	0.245
Monoclinic	3.930	4.001	1.018	β = 91.8	0.252
Rhombohedral	5.522		1	59.80	0

**Table:** (b)

Structure	*a* (Å)	*c* (Å)	c/a	α (degree)	$\Delta E_{\mathrm{tot}}$ (eV/f.u.)
Tetragonal					
zwA-type	3.753	4.533	1.208	90	0.369
zwC-type	3.716	4.596	1.237	90	0.298
zwG-type	3.729	4.546	1.219	90	0.226
Monoclinic	3.777	4.557	1.207	β = 91.4	0.223
Rhombohedral	5.563		1	59.58	0

**Figure 7:**
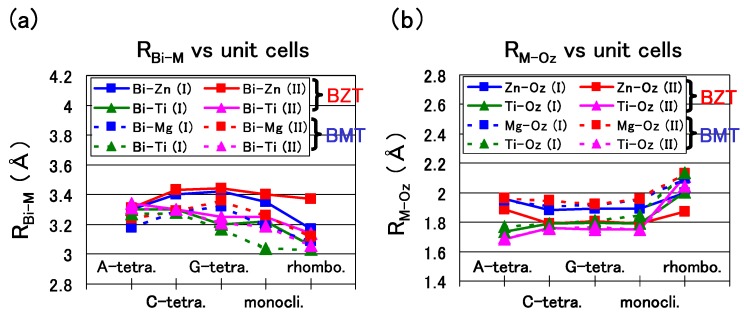
(**a**) $R_{\mathrm{Bi}-\mathrm{Zn}}$, $R_{\mathrm{Bi}-\mathrm{Ti}}$, and $R_{\mathrm{Bi}-\mathrm{Mg}}$ of the A-, C-, and G-type tetragonal, monoclinic, and rhombohedral structures; (**b**) $R_{\mathrm{Zn}-O_{z}}$, $R_{\mathrm{Ti}-O_{z}}$, and $R_{\mathrm{Mg}-O_{z}}$ of the same structures [18].

Let us discuss in detail the optimized results of BZT and BMT in the following. Figure 7(a) shows the nearest distance of Bi-Zn ($R_{\mathrm{Bi}-\mathrm{Zn}}$), Bi–Ti ($R_{\mathrm{Bi}-\mathrm{Ti}}$), and Bi–Mg ($R_{\mathrm{Bi}-\mathrm{Mg}}$). A similar trend is shown in Figure 7(a) except for the $R_{\mathrm{Bi}-\mathrm{Zn}}$ of the rhombohedral BZT in Case II, which is larger than that in Case I. This result must be due to the stronger Coulomb repulsion between Bi and Zn in Case II, and the larger $R_{\mathrm{Bi}-\mathrm{Zn}}$ would make the rhombohedral BZT less stable in Case II. Figure 7(b) shows the nearest distance of Zn–O${}_{z}$ ($R_{\mathrm{Zn}-O_{z}}$), Ti–O${}_{z}$ ($R_{\mathrm{Ti}-O_{z}}$), and Mg–O${}_{z}$ ($R_{\mathrm{Mg}-O_{z}}$). In the C-type, G-type tetragonal, and monoclinic structures of BZT in Case II, whose $\Delta E_{\mathrm{tot}}$ is small, within 0.1 eV/f.u., the $R_{\mathrm{Zn}-O_{z}}$ in Case II is smaller than that in Case I, *i.e*., the Zn ion displacement to O${}_{z}$ in Case II is larger than that in Case I. This result must be due to the stronger Zn–O${}_{x}$ Coulomb repulsion in Case II than that in Case I, as discussed in the case of tetragonal BaTiO${}_{3}$ in the previous subsection. Note that $R_{\mathrm{Zn}-O_{z}}$ and $R_{\mathrm{Ti}-O_{z}}$ of BZT in Case II show similar values. This result seems to be consistent with the experimental result [25] of the random Zn/Ti ordering. The above strong Coulomb repulsion of Bi-Zn and Zn–O of BZT in Case II is due to the 3s${}^{2}$, 3p${}^{6}$, and 3d${}^{10}$ electrons of the Zn ion. Note that the existence of the semicore 3s${}^{2}$ and 3p${}^{6}$ electrons in addition to the 3d${}^{10}$ ones is essential for the strong Coulomb repulsion of the Zn ion.

**Figure 8:**
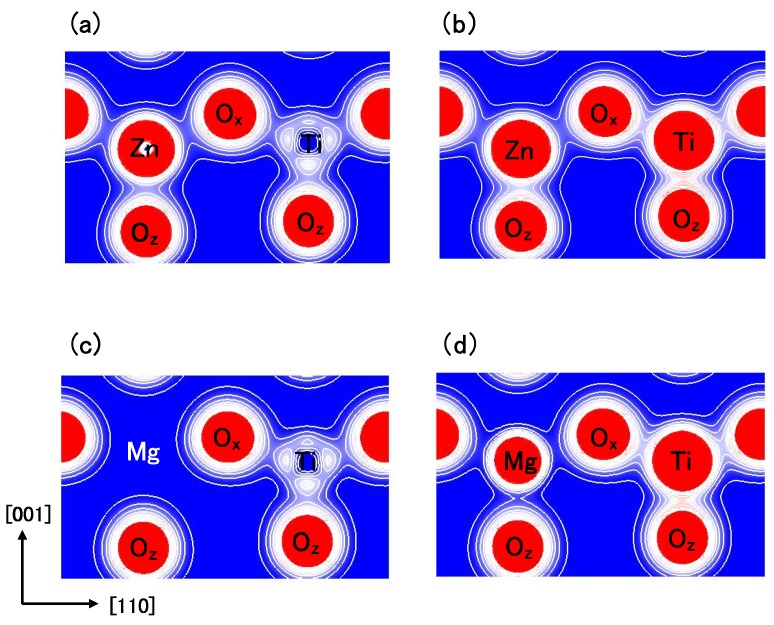
Two-dimensional electron density contour maps of monoclinic (**a**) BZT in Case I; (**b**) BZT in Case II; (**c**) BMT in Case I, and (d) BMT in Case II. The electron density increases as color changes from blue to red via white. Contour curves are drawn from 0.2 to 2.0 *e*/${Å}^{3}$ with increments of 0.2 *e*/${Å}^{3}$ [18].

Figure 8(a) and Figure 8(b) show two-dimensional electron density contour maps of the C-type tetragonal BZT in Cases I and II, respectively. The Coulomb repulsion of Zn–O${}_{x}$ in Case II is larger than that in Case I, and the Coulomb repulsion favorably causes Zn ion displacement to O${}_{z}$ in Case II. This result is consistent with our previous subsection. In contrast to the properties of Zn–O bondings, the inner coordinations of the Ti ion are similar in both cases, although the electron densities are markedly different. This result suggests that the Coulomb repulsion magnitude of Ti–O${}_{z}$ is the same as that of Ti–O${}_{x}$ in small Ti–O bonding (≈ 1.8 Å), in both Cases I and II. Figure 8(c) and Figure 8(d) show two-dimensional electron density contour maps of the C-type tetragonal BMT in Cases I and II, respectively. Although the electron densities in both cases are markedly different, the inner coordinations of the Mg ion are similar. This result suggests that the Coulomb repulsion between Mg and O is not strong sufficiently for inducing Mg ion displacement even in Case II.

**Figure 9:**
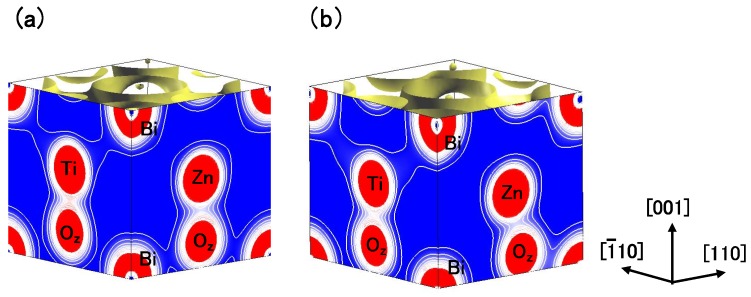
Two-dimensional electron density contour maps of BZT in Case II (**a**) C-type tetragonal and (**b**) monoclinic. The electron density increases as color changes from blue to red via white. Contour curves are drawn from 0.2 to 2.0 *e*/${Å}^{3}$ with increments of 0.2 *e*/${Å}^{3}$ [18].

Finally in this subsection, we discuss the difference in the electronic structures between the C-type tetragonal and the monoclinic BZT. Figure 9(a) and Figure 9(b) show the electron density contour maps of the C-type tetragonal BZT and that of the monoclinic BZT in Case II, respectively. This result suggests that the strong Coulomb repulsion between Zn and O${}_{z}$ causes the small Zn ion displacement in the [$\bar{1}$ $\bar{1}$0] direction in the monoclinic BZT, which makes the Coulomb repulsion of Zn–O${}_{z}$ weaker than that in the C-type tetragonal BZT. As a result, this small Zn ion displacement makes the monoclinic BZT more stable than the C-type tetragonal structure.

## 4. Summary

Using a first-principles calculation with optimized structures, we have investigated the role of the Coulomb repulsions between Ti 3s and 3p states and O 2s and 2p states in ferroelectric BaTiO${}_{3}$. It has been found that the Coulomb repulsions between Ti 3s and 3p${}_{x\;(y)}$ states and O${}_{x\;(y)}$ 2s and 2p${}_{x\;(y)}$ states are closely related to the appearance of Ti ion displacement in tetragonal BaTiO${}_{3}$. This mechanism seems to be consistent with the appearance of Ti ion displacement in rhombohedral BaTiO${}_{3}$. Our present investigation suggests that the Coulomb repulsions between Ti 3s and 3p states and O 2p states have an important role in ferroelectricity. In addition to this suggestion, we believe that our present investigation will show a guideline for the choice of PPs when first-principles calculations with PP methods are performed. We have also investigated the electronic and structural properties of BZT. It has been found that the strong Coulomb repulsion between Zn and O, due to the 3s, 3p, and 3d (d${}^{10}$) states of the Zn ion, favorably causes Zn ion displacement to O ion. The Zn ion also causes the strong Coulomb repulsion to Bi, and the above strong Coulomb repulsion of Zn–O and Bi–Zn makes the pseudo-tetragonal monoclinic or tetragonal structure more stable than the rhombohedral structure. The calculated results show that the pseudo-tetragonal monoclinic structure is more stable than the tetragonal structure. Although this result seems to be in disagreement with the experimental result indicating that the tetragonal structure is the most stable, the calculated lattice constants are in good agreement with the experimental ones. Our investigation suggests that the Coulomb repulsion of Zn–O and Bi–Zn has an important role in the structural stability of BZT. In BMT, on the other hand, the weak Coulomb repulsion of Mg–O and Bi–Mg makes the rhombohedral structure more stable than the tetragonal or monoclinic structure.
